# Evaluation of the German living guideline “Protection against the Overuse and Underuse of Health Care” – an online survey among German GPs

**DOI:** 10.1186/s12875-024-02657-1

**Published:** 2024-12-12

**Authors:** Lisette Warkentin, Martin Scherer, Thomas Kühlein, Felix Pausch, Dagmar Lühmann, Cathleen Muche-Borowski, Susann Hueber

**Affiliations:** 1 Institute of General Practice, Friedrich-Alexander-Universität Erlangen-Nürnberg, Uniklinikum Erlangen, Erlangen, Germany; 2 Institute and Polyclinic for Primary Care and Family Medicine, University Medical Center Hamburg-Eppendorf (UKE), Hamburg, Germany

**Keywords:** Primary care, Guidelines, Overuse, Overdiagnosis

## Abstract

**Background:**

The aim of this study was to evaluate the awareness and use of the German guideline “Protection against the overuse and underuse of health care” from the general practitioners’ (GPs’) perspective. In addition, the study assessed how GPs perceive medical overuse and what solutions they have for reducing it.

**Methods:**

We performed a cross-sectional online survey with recruitment from 15.06. to 31.07.2023. Participants were members of the German College of General Practitioners and Family Physicians (DEGAM). The main outcomes were the awareness and use of the guideline.

**Results:**

The analysis included data from 626 physicians. 51% were female and the median age was 50 years. The guideline is known by 81% of the participants, 32% read it in more detail. The majority considered the guideline a helpful tool in reducing overuse (67%). Almost 90% wished to have more guidelines with clear do-not-do recommendations. Physicians indicated in mean (*M*) that 30.2% (*SD* = 19.3%) of patients ask them for medical services that they do not consider to be necessary and that *M* = 30.2% (*SD* = 18.1%) of all GP services can be attributed to medical overuse. About half of the participants thought that overuse is a moderate or major problem in their practice (52%) and in general practice overall (58%). More participants rated that it is especially a problem in specialist (87%) and inpatient care (82%). Changes in the reimbursement system, raising awareness for the problem and more evidence-based guidelines were considered helpful in mitigating overuse.

**Conclusions:**

Although the guideline is seen as a useful tool in mitigating medical overuse, there is still further potential for its implementation and utilisation. GPs see more overuse in the inpatient and outpatient specialist areas than in their area of practice. Instead of self-critically approaching the problem, the proposed strategies are aimed at the healthcare system itself.

**Supplementary Information:**

The online version contains supplementary material available at 10.1186/s12875-024-02657-1.

## Background

Medical overuse occurs when “a healthcare service is provided under circumstances in which its potential for harm exceeds the possible benefit” [1] and is accompanied by underuse, an insufficient provision of necessary care. The importance of reducing overuse is based on the direct consequences for the patients, e.g. overdiagnosis [2], radiation exposure from X-ray examinations [3] and antibiotic resistance [4]. The ongoing constant growth of healthcare services and costs pose a substantial problem for the systems [5–7]. The healthcare sector seems to be responsible for 5% of the total global environmental impact of industrialised countries [8]. To relieve the healthcare systems, it is of great significance that healthcare resources are being used reasonably and medical overuse is being tackled.

Even though overdiagnosis has already been described in the late nineteenth century under different terms, its criticism became more present in the last years [9]. In 2002, the British Medical Journal published the first theme issue “Too much medicine” of their overdiagnosis series [10], followed by the Journal of the American Medical Association (JAMA) “Less is more” series [11], the “Choosing wisely” campaign and the “Preventing Overdiagnosis Conference” [12, 13].

A survey among German general practitioners (GPs) showed in 2017 that they consider 38% of overall medical services to be overuse [14]. Studies have shown that GPs perceive overuse as a problem. As causes and influencing factors they saw the high patient expectations, lack of time for patient education, fear of liability, economic interests triggered by the remuneration system but also disregard of guidelines [14–16].

In reaction to all this, in June 2019, the German College of General Practitioners and Family Physicians (DEGAM) published the first version of the living-guideline on „Protection against the overuse and underuse of healthcare – deciding together"[17]. The aim of the guideline is to reduce unnecessary interventions and unmet needs in primary care. In the development process of the guideline, „Top-Lists" of recommendations for protection against over- and underuse were extracted in predefined methodological steps from the DEGAM guidelines and national healthcare guidelines (“Nationale Versorgungsleitlinien”). Afterwards, all selected recommendations from the high-quality evidence- and consensus-based guidelines were prioritised by relevance for over- and or underuse by GPs, other healthcare professionals and patient representatives. In the last step, the relevant recommendations were commented on from a GP perspective [18]. In February 2024, the 5th update of the living guideline was published [19].

The aim of this study was to evaluate the awareness and use of the German guideline “Protection against the overuse and underuse of healthcare” [19] from the GPs’ perspective. As this study was carried out in preparation of a workshop at the “Preventing Overdiagnosis Conference" in Copenhagen in 2023 [20], the focus was on medical overuse rather than underuse. In addition, the study assessed how GPs perceive overuse and what solutions they propose for reducing it.

## Methods

In a cross-sectional study design we conducted an online survey among members of the DEGAM. The reporting of the study is based on the STROBE (Strengthening the Reporting of Observational studies in Epidemiology) recommendations [21]. The Ethics Committee of the Friedrich-Alexander Universität Erlangen-Nürnberg approved the study (23–161-S, 08.05.2023).

### Participants and recruitment

Recruitment took place via the DEGAM mailing list. The DEGAM is the scientific college of General Practitioners and Family Physicians in Germany. More than 10% of GPs in Germany are members. All members were invited to participate. The members of the DEGAM are mainly GPs, specialists in internal medicine working as GPs, non-medical researchers, residents, students, and medical assistants. However, this analysis is limited to physicians. The only exclusion criterium was a missing informed consent.

On 15.06.2023 all members of the DEGAM (mailing list with 7.200 e-mail addresses) received an email invitation with a link to the online survey. After giving their informed consent and agreeing to the data protection agreement, the survey started. Withdrawal was possible at any time. Due to the anonymous nature of the survey, it was not possible to delete data after completion. There is a theoretical possibility for multiple attempts. However, as we did not offer any other incentives, we considered this possibility to be rather unlikely. Data collection ended on 31.07.2023. Data were collected using the web-based software platform REDCap (Research Electronic Data Capture), hosted at Uniklinikum Erlangen [22, 23].

### Survey

The first part of the survey focused on physicians’ perceptions of overuse in different areas of healthcare, including an estimate of overuse in their own practice and generally in primary care. The second part assessed the awareness of the guideline. Participants who reported that they read the guideline were asked about its use. These parts were analysed quantitatively. The last content-related question was an open text field to explain from their perspective what would help to reduce overuse and what physicians, professional associations and organisations, but also society (e.g. employers, medical societies, legislators) could do to avoid overuse. This question was analysed qualitatively. Socio-demographic information was collected at the end of the survey. All questions were obligatory (Supplemental Material).

### Analysis

Statistical analyses were performed using SPSS, version 28.0 (IBM Statistics). In the plausibility checks, implausible age was set to missing (0 years, 112 years, *n* = *2*). In case of implausible years of work experience in a GP practice, the variable was set to missing (work experience of 30 years in participants with an age of 34 to 53 years, *n* = 6). The calculated response rate refers to included cases after exclusion in relation to all invitations sent out. Data were first analysed descriptively. Participants who stated that they have read the guideline were compared to those who had only heard of it or who did not know the guideline (readers vs. non-readers) regarding opinion of overuse and where they expect overuse to happen. For the comparison of the opinion of overuse (6-point Likert-scale: 1: I do not agree; 6: I absolutely agree), we report means with standard deviations for each group. A 6-point Likert-scale was used to avoid neutral answers. We performed t-tests for independent samples in case of *p* ≥ 0.05 in Levene's tests of variance homogeneity. In case of statistical significance, Welch’s t-test was used. Cohen’s *d* was calculated as effect size. The questions on where overuse is expected to be a problem (4-point Likert-scale) was transformed into a binary variable (no/minor problem versus moderate/major problem) to descriptively report the results for the two groups. To compare the groups, we used the Mann–Whitney-U test on the 4-point Likert-scale variable. Pearson's correlation coefficient *r* was calculated as effect size. A 2-sided p-value of less than 0.05 was considered statistically significant for all tests.

Answers in the open-text fields were analysed and inductively grouped into several categories. Answers were coded by FP, LW and revised by SH.

### Patient and public involvement

Patients or the public were not involved in the design, conduct, reporting or dissemination plans of this research.

## Results

The response rate was approximately 12%. Questionnaires with missing information on gender or age were excluded from the analysis (*n* = 168). Questionnaires were only included if the physician works in a hospital or practice (excluded *n* = 7). After exclusion, 626 cases remained. Out of the 626 participants who remained after exclusion, 81.8% were GPs, 8.1% were specialists in internal medicine working as GPs, 0.5% were physicians without specialist training and 9.6% were residents. Most of the participants (*n* = 613) were working in a practice, 23 in a hospital and/or 37 in a research institute (e.g. university). The mean age was 49.9 years (SD = 10.8 years) and 50.6% of participants were female. Among the physicians, 19.0% had a work experience in a practice of less than 5 years, 22.9% of 5 to 10 years, 32.5% of 11 to 20 years and 25.0% of more than 20 years (Table 1).

**Table 1:** Sociodemographic characteristics

	***n* or mean**	**% or SD**
**Age (*N* = 624; *NA* = *2) (mean; SD)***	49.9	10.8
**Gender (*N* = 626; *NA* = *0)***	*n*	%
Female	317	50.6
Male	302	48.2
No answer	7	1.1
**Specialisation (*N* = 626; *NA* = *0)***	*n*	%
General practitioner (GP)	512	81.8
Specialist in internal medicine working as GP	51	8.1
Physician without specialist training	3	0.5
Resident	60	9.6
**Work location(s) (multiple answers possible) (*N* = 626; *NA* = *0)***	*n*	%
Practice	613	97.9
Hospital	23	3.7
Research institution (e.g. university)	37	5.9
**Work experience in a GP practice (*N* = 607; *NA* = *19)***	*n*	%
< 5 years	119	19.6
5–10 years	139	22.9
11–20 years	197	32.5
21–30 years	93	15.3
> 30 years	59	9.7
**Location of GP practice (*N* = 613; *NA* = *13)***	*n*	%
Urban	242	39.5
Suburban	143	23.3
Rural	228	37.2
**Practice volume per 3-month interval (*N* = 613; *NA* = *13)***	*n*	%
≤ 1000	257	41.9
1001–1500	272	37.0
> 1500	129	21.0
**Memberships (*N* = 626; *NA* = *0)***	*n*	%
DEGAM	580	92.7
GP association	424	67.7
Local GP association/physicians’ regular meeting	248	39.6
Other association (e.g. Virchowbund, Hartmannbund)	85	13.6
Physicians’ network	91	14.5
MEZIS	55	8.8
Others	113	18.1
None	8	1.3

*SD* Standard deviation, *DEGAM* German Society of General Practice and Family Medicine, *MEZIS* Non-profit organisation

### Awareness and use of the guideline (*N* = 626)

**Fig. 1:**
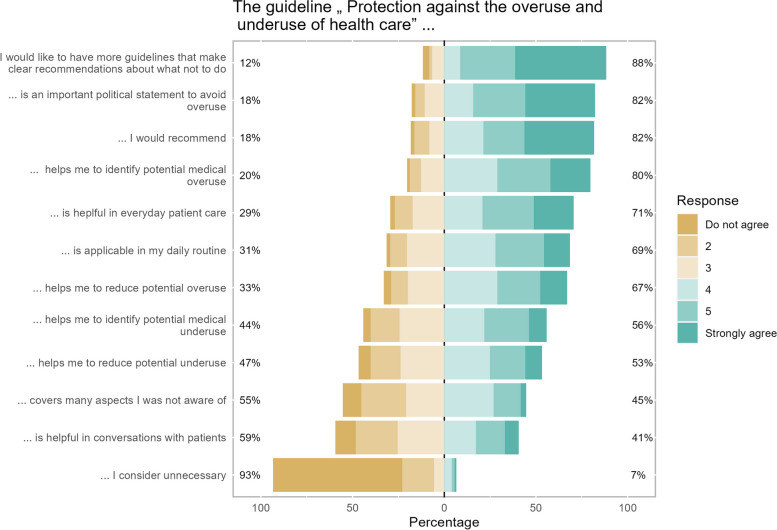
Guideline usability. *N* = 197

Overall, 31.5% (*n* = 197) of the participants had already studied the guideline in more detail and 49.8% (*n* = 312) had heard of it but had not read it yet. Participants who had studied it in more detail were asked about the use of the guideline. The majority agreed that the guideline is an important political statement to avoid overuse (82.2%) and that they would recommend it (81.7%). Most participants considered it helpful in everyday patient care (70.6%) and agreed that it is applicable in their daily routine (68.5%). The majority of participants considered the guideline helpful in identifying (79.7%) or reducing overuse (67.0%). Considerably fewer physicians assessed the guideline helpful in identifying (55.8%) or reducing underuse (53.3%). Less participants (40.6%) stated that the guideline is helpful in conversations with patients or agreed that it covers many aspects they were not aware of (44.7%). Most physicians (88.3%) approved that they want more guidelines that make clear recommendations on what not to do and 6.6% rather considered the guideline unnecessary (Fig. 1).

### Opinion on overuse (*n* = 626)

**Fig. 2:**
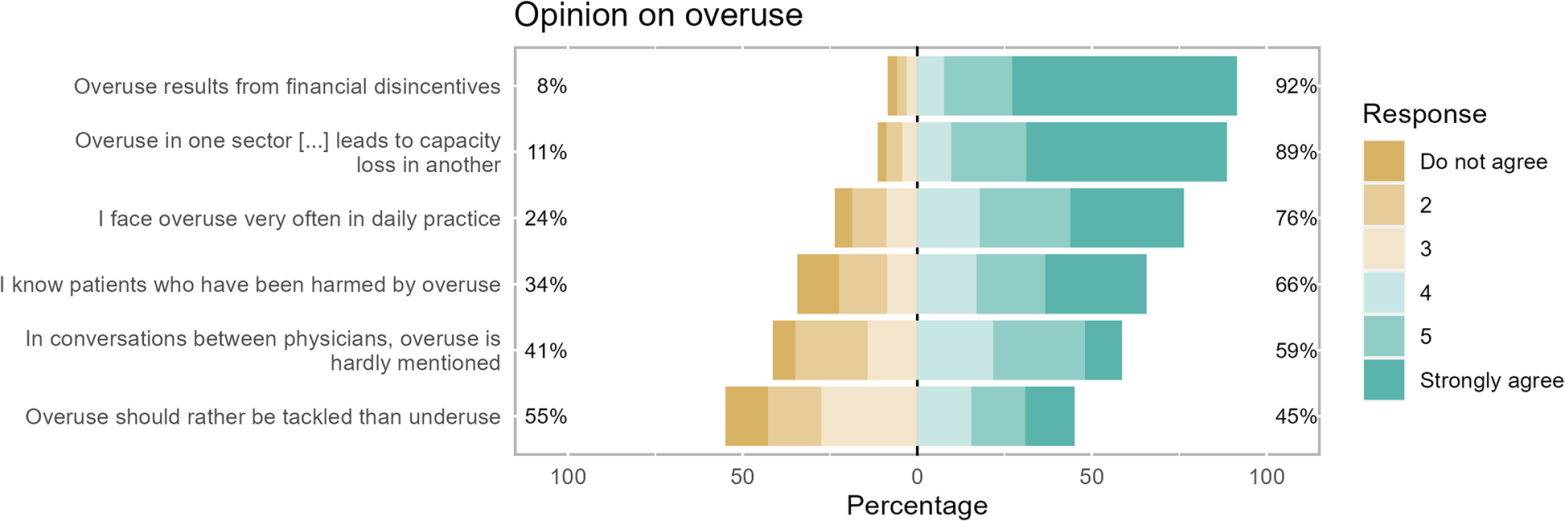
Physicians' opinion on overuse. *N* = 626

The majority of physicians agreed to the statement that overuse results from financial disincentives and that overuse in one sector of the healthcare system leads to capacity loss in another (91.5% and 88.7%). Most of the participants concurred that they face overuse often in their daily practice and that they know patients who have been harmed by overuse (76.4% and 65.7%). More ambiguous was the physicians’ opinions on whether overuse is hardly mentioned in conversations between physicians and whether overuse should rather be tackled than underuse (58.6% and 45.0% agree) (Fig. 2, Table 2).

**Table 2:** Comparison of the opinion on overuse between persons who have read the guideline and those who have not

	**All participants (*N* = 626)**	**Readers (*N* = 197)**	**Non-Readers (*N* = 429)**			
	**Mean (SD)**	**Mean (SD)**	**Mean (SD)**	**Statistics**	***p-*value**	**Effect size (d)**
**Opinion on overuse**^**a**^						
* I face overuse very often in daily practice*	4.47 (1.51)	5.08 (1.1)	4.2 (1.59)	t(527.57) = 8.06^b^	< 0.001	0.61
* I know patients who have been harmed by overuse*	4.06 (1.75)	4.62 (1.52)	3.80 (1.79)	t(444.17) = 5.91^b^	< 0.001	0.48
* In conversations between physicians, overuse is hardly mentioned*	3.73 (1.47)	3.5 (1.43)	3.83 (1.49)	t(624) = −2.57	0.010	−0.22
* Overuse should rather be tackled than underuse*	3.49 (1.57)	3.78 (1.48)	3.36 (1.59)	t(624) = 3.18	0.002	0.27
* Overuse in one sector of the healthcare system leads to capacity loss in another*	5.15 (1.28)	5.4 (1.04)	5.04 (1.36)	t(488.25) = 3.63^b^	< 0.001	0.28
* Overuse results from financial disincentives*	5.32 (1.19)	5.47 (0.88)	5.24 (1.31)	t(538.09) = 2.55^b^	0.011	0.19
* How many of your patients ask you for medical services that you do not consider necessary?*	30.23 (19.29)	32.75 (20.19)	29.08 (18.77)	t(624) = 2.22	0.027	0.19
* How high do you estimate the proportion of overuse of GP services in Germany in relation to all GP services?*	30.24 (18.12)	32.47 (18.76)	29.22 (17.74)	t(624) = 2.09	0.037	0.18
	**%**	**%**	**%**	**Statistics**	***p-*value**	**Effect size (r)**
**Overuse is no/a minor problem, …**^**c**^						
* … in my practice/hospital*	47.9	34.5	54.1	U = 32,138.5 Z = −5.14	< 0.001	−0.21
* … in GP care*	42.0	28.9	48.0	U = 32,402 Z = −5.02	< 0.001	−0.20
* … in outpatient specialised care*	14.1	7.6	17.0	U = 32,199 Z = −5.25	< 0.001	−0.21
* … in inpatient care*	17.7	13.2	19.8	U = 36,733 Z = −2.84	0.005	−0.11
* … in German healthcare system*	8.5	2.0	11.4	U = 33,368.5 Z = −4.8	< 0.001	−0.19
* … in other industrialised nations*	53.4	45.7	56.9	U = 36,524 Z = −2.98	0.003	−0.12

^a^6-point Likert-scale from 1 (Do not agree) to 6 (Strongly agree). ^b^Welch test ^c^Binary variable (moderate/major problem vs. minor/no problem); statistics with 4-point Likert-scale variable

On a scale from 0 to 100%, physicians indicated that around one third of their patients ask them for medical services that they do not consider to be necessary (mean (*M*) = 30.2%; *SD* = 19.3%). Similar to this, participants estimated that about one-third of all medical services provided in primary care are attributed to medical overuse (*M* = 30.2%; *SD* = 18.1%).

### Areas where overuse is expected to be a problem (*n = *626)

**Fig. 3:**
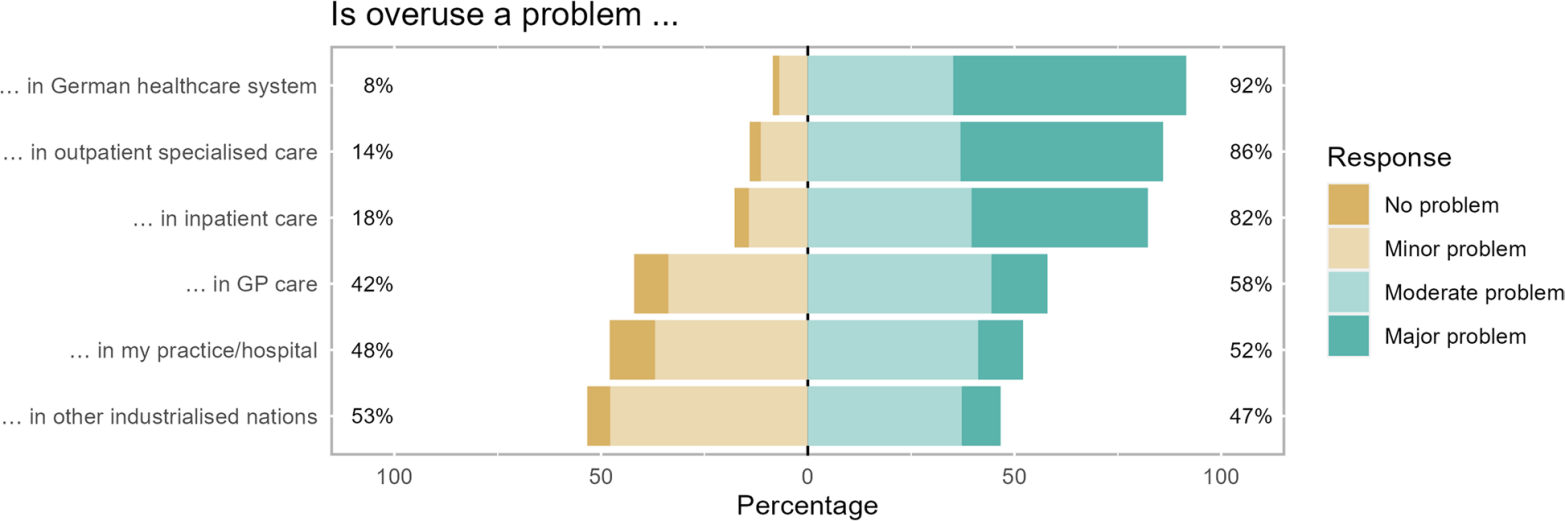
Where is overuse a problem? GP, general practice. *N* = 626

About 52.1% either reported that overuse was a moderate (41.2%) or major problem (10.9%) in their practice/hospital. A little more thought that it was a moderate problem (44.4%) or a major one (13.6%) in general practice overall. Regarding ambulatory specialist care, inpatient care and the German healthcare system as a whole, more participants thought that it was a moderate or major problem (in sum 85.9%; 82.3%; 91.53%, respectively). Almost half of the participants saw overuse in other developed countries as a moderate (37.2%) or major problem (9.4%) (Fig. 3, Table 2).

### Comparison of readers versus non-readers of the guideline

Compared to non-readers, readers of the guideline more often stated that they face overuse and more often know patients who have been harmed by it (*M* (*SD*) = 5.08 (1.10) versus 4.20 (1.59), t(527.57) = 8.06, *p* < 0.001, d = 0.61 and 4.62 (1.52) versus 3.80 (1.79), t(444.17) = 5.91, *p* < 0.001, d = 0.48, respectively). They also more often agreed that overuse should rather be tackled than underuse (3.78 (1.48) versus 3.49 (1.57), t(624) = 3.18, *p* = 0.002, d = 0.27). Non-readers more often reported that in conversations between physicians, overuse is hardly mentioned (3.83 (1.79) versus 3.50 (1.43), t(624) = −2.57, *p* = 0.010, d = −0.22) (Table 2).

Readers of the guideline more often considered overuse to be a problem in their practice, in general practice, in outpatient and inpatient specialised care, in the German healthcare system and in other industrialised nations as well. The difference is statistically significant (*p* = < 0.001 to 0.005) with a low effect size (r = −0.21 to −0.11).

### Measures to mitigate overuse

**Fig. 4:**
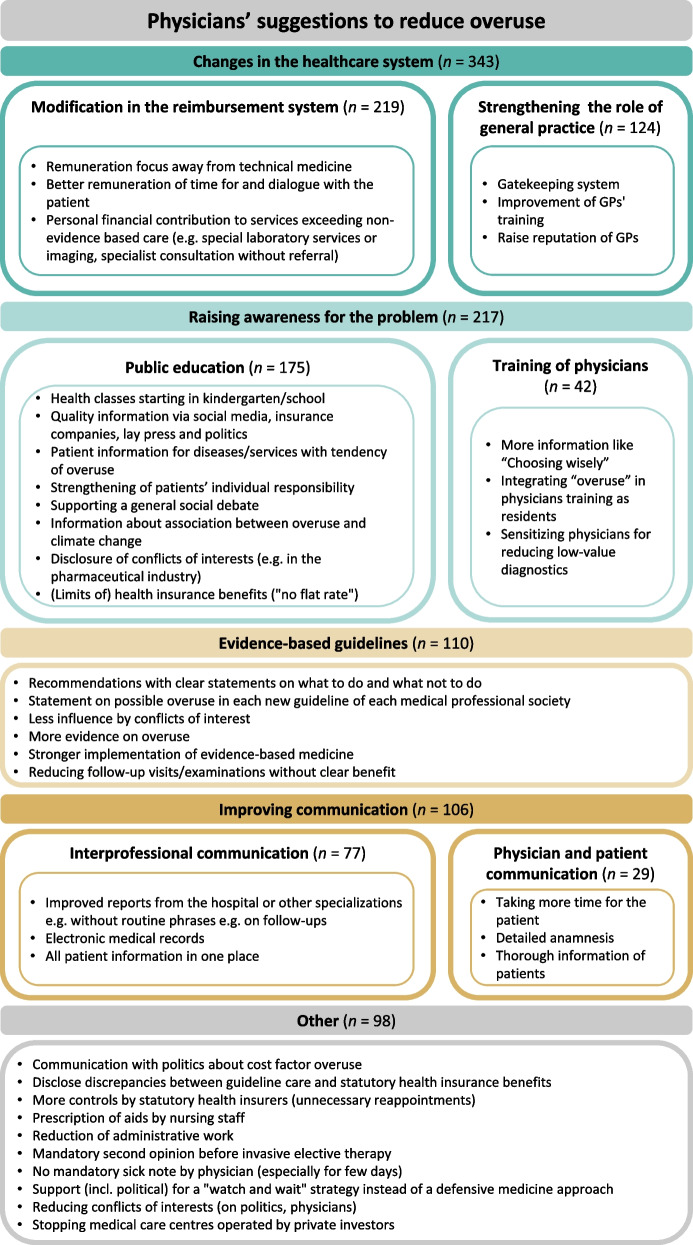
Participants’ suggestions to reduce overuse. Open-text field answers were analysed and inductively grouped into several categories (depicted here with several examples for each category). No statement or statement not concerning the question *n* = 73

Participants named a lot of ideas to reduce overuse. Most could be classified in the following categories: changes to the reimbursement system/reducing financial disincentives, public education, strengthening the role of general practice, guidelines and clear recommendations and improving interprofessional communication. Examples for each category are depicted in Fig. 4.

## Discussion

### Summary of the results

The majority of physicians reported that they are often confronted with overuse and two thirds knew patients who have been harmed by overuse. Nevertheless, only about half of the participants thought that overuse is a moderate or major problem in their own practice and in general practice overall. The participants saw the problem to be bigger in specialist and inpatient care. The guideline “Protection against the overuse and underuse of health care” is known by more than 80% of the participants and around one third read it in more detail. The majority of those considered the guideline a helpful tool in clinical practice and in reducing overuse. Almost all of the participants wished to have more guidelines with clear do-not-do recommendations. Participants who read the guideline rated overuse as a bigger problem than those who had not read it. Physicians suggested that especially changes in the reimbursement system, raising awareness for the problem, more evidence-based guidelines and improving of communication would be helpful in mitigating overuse.

### The use and acceptance of the guideline

The problem of medical overuse was acknowledged by the majority of the physicians in our study. However, participants who had read the guideline considered overuse more often a problem compared to non-readers. With our data we cannot conclude whether the guideline rose the attention on the topic or physicians already primed on this subject read the guideline more often. Howsoever, participating physicians agreed that the guideline can be a helpful tool in mitigating overuse. Most of the physicians would recommend the guideline and almost all of the participants would like to have more guidelines with do-not-do recommendations. In a former study, GPs also agreed that guidelines should explicitly indicate that certain medical services should not be performed [14]. Also, in research and international campaigns do-not-do statements are common [21, 24–26]. Telling physicians what they should not do seems to be interpreted as a facilitation of their workflow and -load, and not as a threat to their professional freedom. Including do-not-do recommendations in guidelines explicitly would strengthen the importance and facilitate the implementation of these recommendations, as the guidelines have the mandate of their respective medical society. Do-not-do recommendations could also be implemented with less financial investment and structural modification than changes in reimbursement or education and training.

### The guideline as a political statement

As much as it is seen as a tool to reduce overuse, the physicians seem to value the guideline for being a political statement. With the development of a GP guideline on the subject of overuse, DEGAM clearly stated the political will that more effort is urgently needed to solve this issue: Too much medicine can be harmful; moreover, overuse and overdiagnostics result in a lack of resources for what is really important [27]. Still, it remains unclear whether the guideline really has an impact on the acknowledgement of medical overuse in politics. Past studies, in line with our results, have shown that system-triggered factors play an important part in upholding medical overuse [28, 29]. For example, financial disincentives are seen as an important driver of medical overuse [15, 29] and changes of the reimbursement system are seen necessary [15, 16, 20]**.** But also, the lack of the GP gatekeeping role is seen as a driver of overuse [14, 16, 30]. In a gatekeeping system, patients need a referral from their GP to see a specialist. Gatekeeping seems to be associated with better quality of healthcare and lower healthcare utilisation and expenditure [31]. In European countries with a gatekeeping system, patient satisfaction with organisational aspects seems to be lower as compared to countries with direct access to specialists. However, this does not seem to impact patient satisfaction regarding the patient-physician relationship [32]. Therefore, this might be a negligible disadvantage. Although mostly healthcare utilisation and expenditure have been studied in regard to gatekeeping systems, Sripa et al. found one study that reported an association between gatekeeping systems and a lower one-year cancer survival [31, 33]. However, the reasons for the observed association remain unclear.

### Other strategies to mitigate overuse

Our participants considered public education and training of physicians important for mitigating overuse. To avoid overuse and raise awareness amongst physicians, various campaigns such as "Choosing Wisely" or its German equivalent "Klug entscheiden" have been launched [12, 34]. A European survey among primary care physician showed that their familiarity varied significantly between countries (79% Sweden vs. 18.1% Greece) [35]. About 50% of German GPs have not heard of any campaign like “Choosing wisely” [14]. Hence, these campaigns possibly need to be advertised more strongly.

In line with our results, other studies concluded that awareness of overuse must be raised, especially among patients [15, 16]. A recent study showed that many people are not familiar with the problem [36]. A qualitative study from the Netherlands with patients who had already been affected by overuse showed that those see overuse e.g. in duplicate care, in care that does not meet their expectations and in inefficient care. Those patients attribute the main causes to poor physician–patient-communication, to care that is not tailored to the individual patient but according to a protocol and to high patients’ expectations [37]. In the past, campaigns targeting the public and healthcare practitioners seemed to be successful. Those campaigns aimed at healthcare issues like reducing antibiotic prescribing [38, 39]. Current projects aim, for example, to reduce the number of sleeping pills [40] or to “bolster the health literacy of the general population by evoking insight that more medicine is not necessarily better for the individual” [41]. One factor in maintaining medical overuse seems to be patients’ demands. Physicians in our study estimated that one third of their patients ask them for services that they do not consider necessary. Patients’ demands are influenced by their intuitive beliefs towards the benefits of interventions. These beliefs include attitudes like more is better, new is better, more expensive is better and technology is good [29]. In addition, both physicians and patients overestimate benefits and underestimate harms of interventions, including cancer screening and medication [42–44]. The belief that more is better has also been demonstrated in a survey in which 56% of citizen agreed that any therapy is better than waiting [45]. These cognitive biases and cultural beliefs underline the importance of further public education and training of physicians.

Our results show that another important aspect is the improvement of the physician–patient communication. From the physicians’ point of view a trusting doctor-patient relationship is crucial for joint decision making [15]. This kind of relationship needs time—a finite resource. If guidelines have the aim to reduce medical overuse and physicians compliantly apply their recommendations, lack of time would be a limiting factor. According to a simulation analysis, providing guideline-recommended primary care (in terms of preventive, chronic disease and acute care for a panel of 2500 patients) would require a 27 h workday [46]. More than half of this time would be spent on preventive care. Therefore, considering physicians’ time needed to treat (TNT) in guidelines has been requested recently [47]. However, the authors state that the “ultimate goals of estimating TNT are to ensure that clinicians and patients spend their limited time together on what is most important for the individual patient and to improve access to care for patients with the greatest care needs”. Providing good care and establishing and maintaining a trusting doctor-patient relationship is time-consuming [48]. However, investing time in good communication could reduce medical overuse and create time for appropriate care in the long term. The do-not-do recommendations can help by providing the physician concrete information and facts so that they can easily justify their decision to patients or other colleagues during the consultation/discussion.

### Overuse seen as a problem for oneself, colleagues and other countries

Medical overuse and low-value care are global healthcare challenges [20, 49]. Braithwaite et al. described our healthcare’s problems in three numbers: 60–30-10, whereas 60% stands for guideline adherent care, 30% for medical overuse and 10% for iatrogenic harm [50]. Physicians in our study estimated in mean a proportion of overuse of 30%, hence exactly what is estimated globally according to Braithwaite et al.. Interestingly, although the estimations match, in our study, more physicians expected that medical overuse is a problem in Germany than in other industrialized nations. Our results show that medical overuse is being acknowledged as a problem in every day patient care in Germany. In our study the participants tended to hold the others as mainly responsible for medical overuse. Other studies have shown similar results [14, 29]. Steinman et al. found a similar relationship for conflict of interests: Most physicians believed that industry promotions and contacts influence the prescribing behaviour of their colleagues, but not their own [51]. A recently published study among European primary care physicians revealed, that the vast majority of them surveyed consider overuse to be a relevant problem in their own healthcare system. Interestingly, they see less overuse in their area of responsibility [35]. In line with these results, the suggested strategies discussed above for mitigating medical overuse mainly apply to others and hardly require any change in behaviour on their own part.

### Limitations and Strengths

This is the first nationwide study evaluating the guideline. Our study population was younger than the average GPs in Germany with more than 70% being older than 50 years in 2022 (versus a median age of 50 years in our population) [52]. However, contrary to this statistic which includes only physicians already specialised in general practice, our population included almost 10% residents. Stewart et al. [53] did not find an association between physicians’ age and guideline adherence [53]. The study population was heterogeneous in terms of work experience, location of the practice and practice volume.

Almost all of our participants were members of the DEGAM (93%). In Germany, there are almost 45.000 physicians specialised in general practice [52]. The DEGAM counted 7.925 members (mainly GPs) in 2023 [54]. The sample of our survey covers therefore only about 1.4% of all GPs in Germany. The DEGAM definition of general practice says that “the aim of general practice is to provide high-quality care, which includes the protection of the patient, but also of the society, from misuse, underuse or overuse of healthcare” [55]. As the DEGAM is a scientific college which is also responsible for the development of new guidelines for GPs in Germany, members probably are more aware of guidelines and the problem of overuse. The sample of our survey is therefore not necessarily representative of GPs in Germany, which limits the generalizability of the findings. The answers regarding the awareness and use of the guideline are based on self-assessment. Therefore, they might be biased towards more positive answers.

## Conclusion

Overuse is acknowledged as a problem by German GPs, at least by those who are members of the DEGAM. The guideline is seen as a useful tool in mitigating medical overuse. However, there is still further potential for its implementation and utilisation. It is considered essential to raise further awareness of the problem among physicians and citizens. GPs attribute overuse mainly to other specialities or inpatient care. If this results from self-serving bias remains unclear. Most of the proposed strategies to tackle overuse are aimed at the healthcare system itself, especially by reducing financial disincentives.

## Supplementary Information

Supplementary Material 1.

Supplementary Material 2.

## Data Availability

The datasets used and analysed during the current study are available from the corresponding author on reasonable request after consent of the data protection supervisor.

## Abbreviations

DEGAM: German College of General Practitioners and Family Physicians
GP: General practitioner
TNT: Time needed to treat
