# Supplementary material for: Evaluation of the German living guideline “Protection against the Overuse and Underuse of Health Care” – an online survey among German GPs
Source: BMC Prim Care. 2024 Dec 12;25:414. doi: 10.1186/s12875-024-02657-1 (PMC11636051; doi:10.1186/s12875-024-02657-1)
Supplement: Supplementary file 1 — Supplementary Material 1. [file 12875_2024_2657_MOESM1_ESM.pdf]

Reducing medical overuse- what can be done?

Dear DEGAM members, dear colleagues,

Thank you for supporting our survey. We are interested in **your perspective and opinion on medical overuse.**

Please find below some important information:

Right of withdrawal: Participation in the survey is **voluntary**. You can end the survey at any time by closing the browser window. However, data that has already been collected cannot be subsequently deleted due to the anonymisation process.

Data protection: Your data will be stored **anonymously** in electronic form on a server at the University Hospital Erlangen. The data is protected against unauthorised access. After completion and evaluation of the study, the anonymised data will be stored for 10 years in accordance with the specifications of the German Research Foundation and then deleted from the server.

The survey takes **about 8 minutes** to complete. Please answer the individual questions spontaneously and according to your personal opinion**. There is no "right" or "wrong" answer.**

Please ensure that you do not use automatic language translation. Experience has shown that errors occur in the presentation of the questionnaire.

I agree to participate in the study and to the storage of my answers:

- ☐ Yes
- ☐ No → **PROG**: End

Reducing medical overuse - what can be done?

Personal information

Recognizing and reducing medical overuse is challenging for patients, therapists and physicians. Please indicate which professional group you belong to?

1. Please state your professional title:

   ☐ 1 General practitioner → **PROG**: continue to 1.2.

   ☐ 2 Specialists in internal medicine working in general practice → **PROG**: continue to 1.2.

   ☐ 3 Physician without specialist training → **PROG**: continue to 1.2.

   ☐ 4 Resident → **PROG**: continue to 1.2.

   ☐ 5 Medical assistant → **PROG**: continue to 1.2.

   ☐ 6 Non-medical researcher at a university

   ☐ 7 Student

   ☐ 8 Other → **PROG**: continue to 1.1.

   1.1. Please specify: _____________

   1.2. Please state in which field you are currently working → **PROG:** Multiple answers possible:

   ☐ 1 In the hospital

   ☐ 2 In a practice

   ☐ 3 Researcher in a research institution/university

Reducing medical overuse - what can be done?

Relevance of medical overuse

1. **Medical overuse** refers to the provision of medical services that are not indicated or whose (net) benefit is not sufficiently confirmed.

There are different opinions on the relevance of medical overuse. What is your point of view?

Below you will find various statements. Please indicate how much you agree with them.

	I do not agree					I absolutely agree
	1	2	3	4	5	6
I face overuse very often in daily practice → **PROG** only if question 1.2 = 1 or 2 (physician in practice or hospital)	O	O	O	O	O	O
In conversations between physicians, overuse is hardly mentioned	O	O	O	O	O	O
Overuse should rather be tackled than underuse	O	O	O	O	O	O
I know patients who have been harmed by overuse	O	O	O	O	O	O
Overuse in one sector of the healthcare system leads to capacity loss in another	O	O	O	O	O	O
Overuse results from financial disincentives	O	O	O	O	O	O

Reducing medical overuse - what can be done?

Relevance of medical overuse

2. To what extent do you consider medical overuse to be a problem in your practice/hospital, in the healthcare system and at national and international level?

   Please complete the sentences by selecting one of the possible answers.

	Not a problem	A minor problem	A moderate problem	A major problem
In **my practice/hospital**, medical overuse is ... → **PROG** only if question 1.2 = 1 or 2 (physician in practice or hospital)	O	O	O	O
In GP care, medical overuse is …	O	O	O	O
In outpatient specialised care medical overuse is…	O	O	O	O
In inpatient care, medical overuse is …	O	O	O	O
In the German healthcare system, medical overuse is ...	O	O	O	O
In other industrialised nations, medical overuse is ...	O	O	O	O

3. → **PROG**: Only if question 1.2 = 1 or 2 (physician in practice or hospital)

   Thinking about an average week in your practice: How many of your patients do you ask for medical services that you do not consider necessary?

   Answer option: Range from 0 to 100 %

4. How high do you estimate the proportion of medical services provided in primary care in Germany that can be attributed to medical overuse?

   Answer option: Range from 0 to 100 %

Reducing medical overuse - what can be done?

DEGAM guideline "Protection against Over- and Underuse of Healthcare"

In the DEGAM guideline "Protection against Over- and Underuse of Healthcare", a list of selected recommendations was compiled on the basis of existing evidence- and consensus-based guidelines to help effectively counteract medical overuse and underuse. The aim is to avoid unnecessary measures and reduce underuse in GP care.

5. Are you familiar with the guideline "Protection against Over- and Underuse of Healthcare"?
   - ☐ Yes, and I have already taken a closer look at it → **PROG:** Go to 6.
   - ☐ I have heard of it, but I have not read the guideline yet → **PROG:** Go to 7.
   - ☐ No -> **PROG:** Go to 7.

6. → <u>**PROG:**</u> Only if question 1.2 = 1 or 2 (physician in practice or hospital)

We are interested in your opinion on this guideline. Please indicate how strongly you agree with the following statements.

		I do not agree					I absolutely agree
1	The guideline is helpful in everyday patient care	O	O	O	O	O	O
2	The guideline is an important political statement to avoid medical overuse	O	O	O	O	O	O
3	The guideline is applicable in my daily routine	O	O	O	O	O	O
4	The guideline helps me to **identify** potential medical overuse	O	O	O	O	O	O
5	The guideline helps me to **reduce** potential medical overuse	O	O	O	O	O	O
6	The guideline helps me to **identify** potential <u>medical underuse</u>	O	O	O	O	O	O
7	The guideline helps me to **reduce** potential <u>medical underuse</u>	O	O	O	O	O	O
8	I would recommend the guideline	O	O	O	O	O	O
9	The guideline covers many aspects that I was not aware of	O	O	O	O	O	O
10	The guideline is helpful in conversations with patients	O	O	O	O	O	O
11	I would like to have more guidelines that make clear recommendations about what not to do	O	O	O	O	O	O
12	I consider the guideline to be unnecessary	O	O	O	O	O	O

→ **PROG:** Only if question 1.1. = 5, 6, 7 or 8 (= medical assistant, scientist, student, other) OR if question 1.2. is ONLY 3 (physicians working only in research).

Do you have any assumptions about how this guideline is used and assessed by physicians in patient care?

Please indicate how much you agree with the following statements.

		I do not agree					I absolutely agree
1	The guideline is helpful for physicians in everyday patient care	O	O	O	O	O	O
2	The guideline is an important political statement to avoid medical overuse	O	O	O	O	O	O
3	The guideline is applicable in physicians' daily routine	O	O	O	O	O	O
4	The guideline helps physicians to **identify** potential medical overuse	O	O	O	O	O	O
5	The guideline helps physicians to **reduce** potential medical overuse	O	O	O	O	O	O
6	The guideline helps physicians to **identify** potential medical underuse	O	O	O	O	O	O
7	The guideline helps physicians to **reduce** potential medical underuse	O	O	O	O	O	O
8	Physicians are likely to recommend this guideline to their colleagues	O	O	O	O	O	O
9	The guideline covers many aspects that physicians were not aware of	O	O	O	O	O	O
10	This guideline is probably helpful in conversations with patients	O	O	O	O	O	O
11	There should be more guidelines that make clear recommendations on what not to do	O	O	O	O	O	O
12	I suspect that many physicians will find this guideline unnecessary	O	O	O	O	O	O

Reducing medical overuse - what can be done?

Your personal opinion on how to avoid medical overuse

We are keen to find out what you would consider doing to avoid medical overuse. Your answers will provide us with important information on how an improvement could be achieved.

7. In your opinion, what are services that contribute to medical overuse and that should be reduced or avoided?

8. → **PROG:** Only if question 1.2 = 1 or 2 (physician in practice or hospital)

What would help you to reduce medical overuse?

Please describe specifically what would encourage you to reduce medical overuse. What could your colleagues, professional associations and organisations, but also society (e.g. employers, medical societies, legislators) do to support you in this?

9. → **PROG:** Only if question 1.1. = 5, 6, 7 or 8 (= medical assistant, scientist, student, other) OR if question 1.2. is ONLY 3 (physicians working only in research)

Which measures do you think would help to reduce medical overuse?

What could physicians, professional associations and organisations, but also society (e.g. employers, medical societies, legislators) do to avoid medical overuse?

Sociodemographic information

Finally, we would like to ask you for a couple of brief details about yourself.

1. Please state your age [in years]:

2. Please state your gender:
   - ☐ male
   - ☐ female
   - ☐ diverse

3. → **PROG**: Only if question 1.2 = 2 (physician in practice)
   How long have you been working in a GP practice?
   - ☐ Less than 5 years
   - ☐ 5 to 10 years
   - ☐ 11 to 20 years
   - ☐ 21 to 30 years
   - ☐ Over 30 years

4. → **PROG**: Only if question 1.2 = 2 (physician in practice)
   Please indicate the area in which your practice is located?
   - ☐ Urban area
   - ☐ Suburban area
   - ☐ Rural area

5. → **PROG**: Only if question 1.2 = 2 (physician in practice)
   On average, how many patients are seen *per physician* in your practice per quarter? Please make a rough estimate.
   - ☐ Up to 500
   - ☐ 501-1000
   - ☐ 1001-1500
   - ☐ More than 1500

6. Are you a member of one of the following organisations/societies?

- ☐ German Society for General Practice and Family Medicine (DEGAM)
- ☐ General practitioners' association
- ☐ Local general practitioners' association / doctors' get-together / service groups
- ☐ Other association (e.g. Ärztebund, Virchowbund, Hartmannbund,…)
- ☐ Network of physicians
- ☐ MEZIS
- ☐ Others → PROG: Continue to 6.1
- ☐ I am not a member of any organisation/society

6.1 Please specify: _____________

End of survey

Thank you very much for participating in our survey!

Please use the following field if you have any questions or would like to comment on the survey. We appreciate any feedback.
