# Supplementary material for: Evaluation of the German living guideline “Protection against the Overuse and Underuse of Health Care” – an online survey among German GPs
Source: BMC Prim Care. 2024 Dec 12;25:414. doi: 10.1186/s12875-024-02657-1 (PMC11636051; doi:10.1186/s12875-024-02657-1)
Supplement: Supplementary file 2 — Supplementary Material 2. [file 12875_2024_2657_MOESM2_ESM.pdf]

STROBE Statement—checklist of items that should be included in reports of observational studies

	Item No.	Recommendation	Relevant text from manuscript
**Title and abstract**	1	(*a*) Indicate the study's design with a commonly used term in the title or the abstract	Title/Abstract
		(*b*) Provide in the abstract an informative and balanced summary of what was done and what was found	Abstract
		**Introduction**	
Background/rationale	2	Explain the scientific background and rationale for the investigation being reported	Background
Objectives	3	State specific objectives, including any prespecified hypotheses	Background
		**Methods**	
Study design	4	Present key elements of study design early in the paper	Methods
Setting	5	Describe the setting, locations, and relevant dates, including periods of recruitment, exposure, follow-up, and data collection	Methods: Participants and recruitment
Participants	6	(*a*) *Cohort study*—Give the eligibility criteria, and the sources and methods of selection of participants. Describe methods of follow-up	NA
		(*b*) *Cohort study*—For matched studies, give matching criteria and number of exposed and unexposed	NA
Variables	7	Clearly define all outcomes, exposures, predictors, potential confounders, and effect modifiers. Give diagnostic criteria, if applicable	Methods: Survey
Data sources/ measurement	8*	For each variable of interest, give sources of data and details of methods of assessment (measurement). Describe comparability of assessment methods if there is more than one group	Methods
Bias	9	Describe any efforts to address potential sources of bias	Methods: Statistical analysis
Study size	10	Explain how the study size was arrived at	Methods: Statistical analysis

Continued on next page

Quantitative variables	11	Explain how quantitative variables were handled in the analyses. If applicable, describe which groupings were chosen and why	Methods: Statistical analysis
Statistical methods	12	(*a*) Describe all statistical methods, including those used to control for confounding	Methods: Statistical analysis
		(*b*) Describe any methods used to examine subgroups and interactions	Methods: Statistical analysis
		(*c*) Explain how missing data were addressed	Methods: Statistical analysis
		(*d*) *Cohort study*—If applicable, explain how loss to follow-up was addressed	NA
		(*e*) Describe any sensitivity analyses	NA
		**Results**	
Participants	13*	(a) Report numbers of individuals at each stage of study—eg numbers potentially eligible, examined for eligibility, confirmed eligible, included in the study, completing follow-up, and analysed	Methods: Statistical analysis
		(b) Give reasons for non-participation at each stage	Methods: Statistical analysis
		(c) Consider use of a flow diagram	NA
Descriptive data	14*	(a) Give characteristics of study participants (eg demographic, clinical, social) and information on exposures and potential confounders	Results + Table 1
		(b) Indicate number of participants with missing data for each variable of interest	Tables/Figures
		(c) *Cohort study*—Summarise follow-up time (eg, average and total amount)	NA
Outcome data	15*	*Cohort study*—Report numbers of outcome events or summary measures over time	NA
Main results	16	(*a*) Give unadjusted estimates and, if applicable, confounder-adjusted estimates and their precision (eg, 95% confidence interval). Make clear which confounders were adjusted for and why they were included	Results + Tables 2 and Figures
		(*b*) Report category boundaries when continuous variables were categorized	Table 1
		(*c*) If relevant, consider translating estimates of relative risk into absolute risk for a meaningful time period	NA

Continued on next page

Other analyses	17	Report other analyses done—eg analyses of subgroups and interactions, and sensitivity analyses	NA
**Discussion**			
Key results	18	Summarise key results with reference to study objectives	Discussion: Summary of the results
Limitations	19	Discuss limitations of the study, taking into account sources of potential bias or imprecision. Discuss both direction and magnitude of any potential bias	Limitations
Interpretation	20	Give a cautious overall interpretation of results considering objectives, limitations, multiplicity of analyses, results from similar studies, and other relevant evidence	Discussion, Limitations, Conclusion
Generalisability	21	Discuss the generalisability (external validity) of the study results	Discussion, Limitations
**Other information**			
Funding	22	Give the source of funding and the role of the funders for the present study and, if applicable, for the original study on which the present article is based	Funding statement

*Give information separately for cases and controls in case-control studies and, if applicable, for exposed and unexposed groups in cohort and cross-sectional studies.

Note: An Explanation and Elaboration article discusses each checklist item and gives methodological background and published examples of transparent reporting. The STROBE checklist is best used in conjunction with this article (freely available on the Web sites of PLoS Medicine at http://www.plosmedicine.org/, Annals of Internal Medicine at http://www.annals.org/, and Epidemiology at http://www.epidem.com/). Information on the STROBE Initiative is available at www.strobe-statement.org.
